# A Molecular Electron Density Theory Study of the Chemoselectivity, Regioselectivity, and Diastereofacial Selectivity in the Synthesis of an Anticancer Spiroisoxazoline derived from α-Santonin

**DOI:** 10.3390/molecules24050832

**Published:** 2019-02-26

**Authors:** Luis R. Domingo, Mar Ríos-Gutiérrez, Nivedita Acharjee

**Affiliations:** 1 Department of Organic Chemistry, University of Valencia, Dr. Moliner 50, Burjassot, E-46100 Valencia, Spain; rios@utopia.uv.es; 2 Department of Chemistry, Durgapur Government College, J. N. Avenue, Durgapur-, West Bengal 713214, India

**Keywords:** α-santonin, nitrile oxides, [3 + 2] cycloaddition reactions, regioselectivity, diastereofacial selectivity, molecular electron density theory

## Abstract

The [3 + 2] cycloaddition (32CA) reaction of an α-santonin derivative, which has an exocyclic C–C double bond, with *p*-bromophenyl nitrile oxide yielding only one spiroisoxazoline, has been studied within the molecular electron density theory (MEDT) at the MPWB1K/6-311G(d,p) computational level. Analysis of the conceptual density functional theory (CDFT) reactivity indices and the global electron density transfer (GEDT) account for the non-polar character of this *zwitterionic-type* 32CA reaction, which presents an activation enthalpy of 13.3 kcal·mol^−1^. This 32CA reaction takes place with total *ortho* regioselectivity and *syn* diastereofacial selectivity involving the exocyclic C–C double bond, which is in complete agreement with the experimental outcomes. While the C–C bond formation involving the β-conjugated carbon of α-santonin derivative is more favorable than the C–O one, which is responsible for the *ortho* regioselectivity, the favorable electronic interactions taking place between the oxygen of the nitrile oxide and two axial hydrogen atoms of the α-santonin derivative are responsible for the *syn* diastereofacial selectivity.

## 1. Introduction

Spiroheterocycles are an important subset of heterocyclic compounds representing an attractive synthetic target due to their biological activity as a consequence of the steric constraints generated by the fusion of two rings at a common point [1,2,3]. Spiroheterocycles, which are key intermediates for generating further molecular complexity, are synthesized by [3 + 2] cycloaddition (32CA) reactions of a three-atom-component (TAC) with an exocyclic alkene [4].

In recent years, several researchers have focused their attention on the synthesis of biologically active spiroisoxazoline derivatives from different plant products, which are less toxic and more reactive candidates for the synthesis of spiroheterocycles [5,6,7,8]. These reactions are quite promising from the stereoselective point of view, and the synthesized spiroheterocycles have shown excellent anticancer properties [5,6].

**Chart 1:**
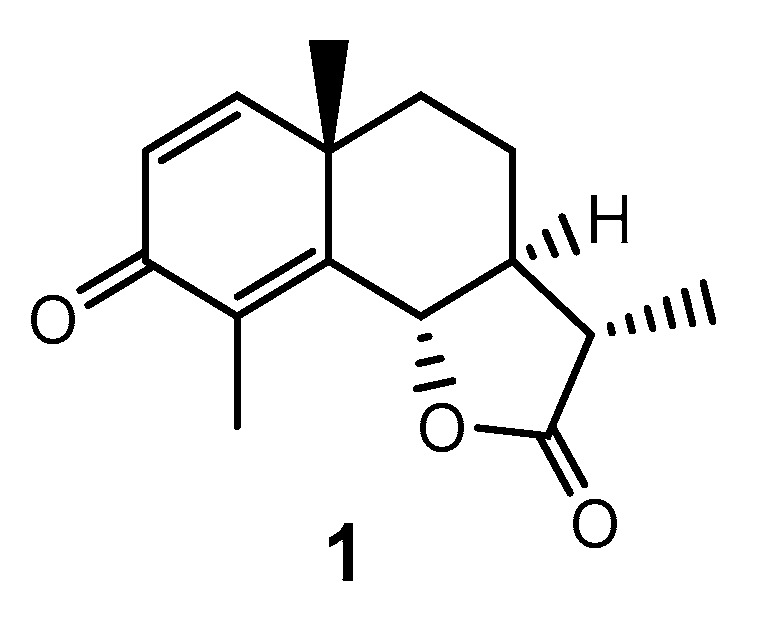
Structure of α-santonin **1**.

α-Santonin **1** is a compound isolated from the flowers of the Artemisia genus plants found in Russia and central Asia that is popularly used as an anthelminthic [9], and is also an important sesquiterpenoid lactone possessing the eudesmane skeleton, which contains a highly functionalized ring with a cross-conjugated dienone system and a lactone moiety (see Chart 1).

**Scheme 1:**
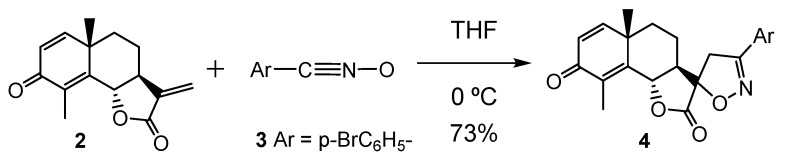
[3 + 2] cycloaddition (32CA) reaction of α-santonin derivative **2** with *p*-bromophenyl nitrile oxide **3**.

In 2013, Kumar synthesized spiroisoxazolidine and spiroisoxazoline derivatives via 32CA reactions of α-santonin derivative **2** with nitrones and nitrile oxides, which showed significant anticancer activity against human cancer cell lines [10]. Synthon **2** was prepared introducing the exocyclic double bond in α-santonin **1**. These 32CA reactions were completely regioselective, leading to the *ortho* isomer as the only cycloadduct. Thus, when *p*-bromophenyl nitrile oxide **3** was used, spiroisoxazoline derivatives **4**, which showed a comparatively more potent IC50 value against HCT-1, PC-3, and MCF-7 cancer cell lines with respect to other substituted cycloadducts, was obtained with 73% yield as the only product through total *ortho* regioselectivity and *syn* diastereofacial selectivity (see Scheme 1).

The mechanism and regioselectivity in 32CA reactions have been extensively investigated on a theoretical level. Numerous recent molecular electron density theory [11] (MEDT) studies of 32CA reactions have allowed establishing a very good correlation between the electronic structure of the simplest TACs and their reactivity [12]. Accordingly, depending on the electronic structure of the simplest TACs, 32CA reactions have been classified into *pseudodiradical-type* (*pdr-type*), *pseudoradical-type* (*pmr-type*), *zwitterionic-type* (*zw-type),* and *carbenoid-type* (*cb-type*) reactions. The reactivity of TACs decreases in the order *pseudodiradical* > *pseudoradical* ≈ carbenoid > zwitterionic. Nitrile oxides are lineal propargylic TACs, which have a zwitterionic structure participating in *zw-type* [12].

**Scheme 2:**
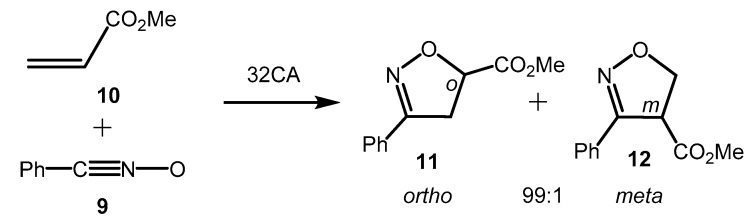
32CA reaction of benzonitrile oxide **9** with methyl acrylate **10**.

The non-polar *zw-type* 32CA reaction of benzonitrile oxide **9** with methyl acrylate **10** was recently studied within the MEDT at the B3LYP/6-31G(d) computational level (see Scheme 2) [13]. This non-polar 32CA reaction has an activation energy in dichloromethane of 12.3 kcal·mol^−1^ and shows poor *ortho* regioselectivity given that the transition state (TS) associated with the formation of *meta* isoxazoline **12** is found to be only 1.6 kcal·mol^−1^ less favorable than that associated with the formation of *ortho* isoxazoline **11** [13].

The synthesis of spiroheterocycles from plant products generate stereospecific cycloadducts, the steric effects and electronic constraints of the reactants being the key factor controlling these reactions. The mechanism of such reactions demands a theoretical rationalization to discern the preferred regioisomeric and stereoisomeric pathway. Consequently, the 32CA reaction of α-santonin derivative **2** with *p*-bromophenyl nitrile oxide **3**, which was experimentally reported by Kumar [10], is herein studied within the MEDT at the MPWB1K/6-311G(d,p) computational level in order to understand the origin of the chemoselectivity, regioselectivity, and diastereofacial selectivity found in the synthesis of spiroisoxazoline **4** (see Scheme 1).

## 2. Computational Methods

Density functional theory (DFT) calculations were performed using the MPWB1K functional [14] together with the 6-311G(d,p) basis set [15]. The suitability of this DFT computational level for the study of *zw-type* 32CA reactions was recently stressed by CCSD(T)/cc-pVTZ single-point energy calculations [16]. Optimizations were carried out using the Berny analytical gradient optimization method [17,18]. The stationary points were characterized by frequency computations in order to verify that TSs have one and only one imaginary frequency. The intrinsic reaction coordinate [19] (IRC) paths were traced in order to check the energy profiles connecting each TS to the two associated minima of the proposed mechanism using the second order González–Schlegel integration method [20,21]. The solvent effects of tetrahydrofuran (THF) were taken into account by full optimization of the gas phase structures using the polarizable continuum model (PCM) developed by Tomasi et al. [22,23] in the framework of the self-consistent reaction field (SCRF) [24,25,26]. Values of enthalpies, entropies, and Gibbs free energies were calculated with standard statistical thermodynamics [15] at reaction conditions: 273.15 K and 1 atm in THF [10].

The global electron density transfer [27] (GEDT) was computed by the sum of the natural atomic charges (q), which were obtained by a natural population analysis (NPA), [28,29], and in which the atoms belonged to each framework (f) at the TSs; i.e., GEDT (f) = $\sum_{q\in f}q$. The sign indicates the direction of the electron density flux in such a manner that positive values mean a flux from the considered framework to the other one. Conceptual DFT (CDFT) global reactivity indices [30,31] and Parr functions [32] were computed using the equations given in reference 31. All of the computations were carried out with the Gaussian 16 suite of programs [33].

Topological analysis of the electron localization function (ELF) [34] was performed with the TopMod [35] package using the corresponding gas phase monodeterminantal wavefunctions. The characterization of the bond formation processes along the most favorable reaction path was carried out by performing the topological analysis of the ELF for some selected structures along the IRC profile. The topological analysis of the non-covalent interactions (NCI) [36] was performed with the NCIplot program [37] by evaluating the gas phase SCF density. The interacting quantum atoms (IQA) energy decomposition analysis [38] was performed with the Promolden program [39] using the gas phase B3LYP/6-31G(d) monodeterminantal wavefunctions of smaller models of the corresponding TSs without optimization, in order to keep the original molecular geometry of the bigger experimental system.

## 3. Results and Discussion

The present MEDT study has been divided into six sections. First, in Section 3.1, an ELF topological analysis of α-santonin derivative **2** and *p*-bromophenyl nitrile oxide **3** is performed in order to determine their electronic structure. Then, in Section 3.2, an analysis of the CDFT reactivity indices of these reagents is carried out. In Section 3.3, the energy profile associated with the 32CA reaction between α-santonin derivative **2** and *p*-bromophenyl nitrile oxide **3** yielding spiroisoxazoline **4** is studied. Next, the origin of the *ortho* regioselectivity and *syn* diastereofacial selectivity along the 32CA reactions of α-santonin derivative **2** with *p*-bromophenyl nitrile oxide **3** is analysed in Section 3.4 and Section 3.5, respectively. Finally, an ELF topological analysis of the C–C and C–N bond formation along the most favorable reaction path is performed in Section 3.6.

### 3.1. ELF Topological Analysis of α-Santonin Derivative **2** and p-Bromophenyl Nitrile Oxide **3**

**Figure 1:**
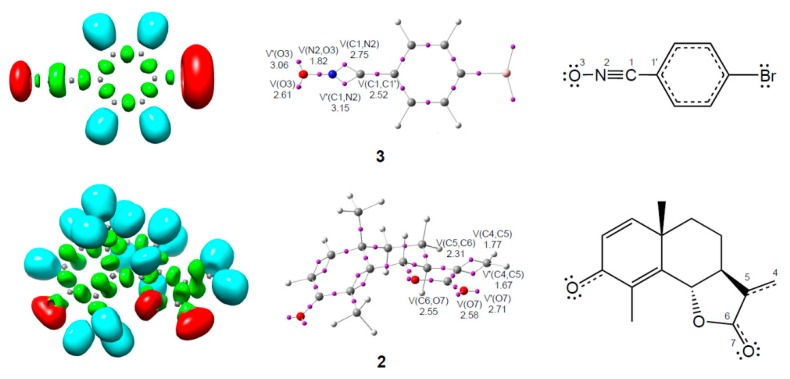
MPWB1K/6-311G(d,p) localization domains (isovalue = 0.75) and basin attractor positions together with the most significant valence basin populations, and the proposed Lewis-like structures of α-santonin derivative **2** and *p*-bromophenyl nitrile oxide **3**. Electron localization function (ELF) valence basin populations are given in average number of electrons, monosynaptic valence basins are shown in red, disynaptic valence basins are shown in green, protonated basins are shown in blue, and core basins are shown in grey.

An appealing procedure that provides a straightforward connection between the electron density distribution and the chemical structure is the quantum chemical analysis of Becke and Edgecombe’s ELF [34]. Therefore, in order to characterize the electronic structures of α-santonin derivative **2** and *p*-bromophenyl nitrile oxide **3** and, thus predict and understand their reactivity in 32CA reactions [12], a topological analysis of the ELF of both reagents was first performed. ELF localization domains and basin attractor positions, together with the most significant valence basin populations, as well as the proposed Lewis-like structures, are shown in Figure 1.

ELF topological analysis of the lactone framework of α-santonin derivative **2** shows the presence of two V(C4,C5) and V′(C4,C5) disynaptic basins integrating a total population of 3.41 e, a V(C5,C6) disynaptic basin integrating 2.31 e, a V(C6,O7) disynaptic basin integrating 2.55 e, and two V(O7) and V′(O7) monosynaptic basins integrating a total population of 5.29 e. On the other hand, an ELF topological analysis of *p*-bromophenyl nitrile oxide **3** shows the presence of a V(C1,C1′) disynaptic basin integrating 2.52 e, two V(C1,N2) and V′(C1,N2) disynaptic basins integrating a total population of 5.90 e, a V(N2,O3) disynaptic basin integrating 1.82 e, and two V(O3) and V′(O3) monosynaptic basins integrating a total population of 5.64 e.

Within the ELF context, monosynaptic basins, labeled V(A), are associated with non-bonding regions, i.e., lone pairs or *pseudoradical* centers, while disynaptic basins, which are labeled V(A,B), connect the core of two nuclei A and B and, thus correspond to a bonding region between A and B [29]. This description, together with the ELF valence basin populations, recovers Lewis’s bonding model, providing a very suggestive graphical representation of the molecular system. Therefore, within Lewis’s bonding model [40], the two V(C4,C5) and V′(C4,C5) disynaptic basins can be associated with an underpopulated C4–C5 double bond, while the V(C5,C6) disynaptic basin would correspond to a slightly overpopulated single bond. In the case of *p*-bromophenyl nitrile oxide **3**, the two V(C1,N2) and V′(C1,N2) disynaptic basins can be related to a C1–N2 triple bond, while the V(N2,O3) disynaptic basin can be related to a somewhat depopulated N2–O3 single bond.

The C4–C5 bonding region of the α,β-unsaturated ketone of *α*-santonin derivative **2** can be considered an underpopulated exocyclic double bond that is only slightly conjugated with the carbonyl group, and thus will exhibit the common reactivity of alkenes. However, the presence of neither *pseudoradical* nor carbenoid centers at *p*-bromophenyl nitrile oxide **3**, and the presence of a C1–N2 triple bond instead, indicates that this linear TAC possesses a propargylic, zwitterionic electronic structure that enables its participation in *zw-type* 32CA reactions only [12].

### 3.2. Analysis of the CDFT Reactivity Indices of the Reagents

Numerous studies devoted to Diels–Alder and 32CA reactions have shown that the analysis of the reactivity indices defined within CDFT [30,31] is a powerful tool for characterizing the non-polar or polar character of a reaction, and thus its feasibility. Consequently, the global indices, namely, the electronic chemical potential (µ), chemical hardness (η), global electrophilicity (ω), and global nucleophilicity (*N*) of α-santonin derivative **2** and *p*-bromophenyl nitrile oxide **3**, were computed and analyzed (see Table 1). The global indices of the simplest nitrile oxide **13** and 3-methylene lactone **16** are also included.

As shown in Table 1, the electronic chemical potential [41] µ of α-santonin derivative **2**, µ = −4.16 eV, is only slightly lower than that of *p*-bromophenyl nitrile oxide **3**, µ = −4.03 eV, suggesting that neither of them will transfer electron density to the other.

Both reagents present similar electrophilicity [42] ω indices of 1.76 eV (**2**) and 1.69 eV (**3**), as well as similar nucleophilicity [43] *N* indices of 2.51 eV (**2**) and 2.69 eV (**3**), being classified as strong electrophiles and moderate nucleophiles [31]. These values, together with the corresponding electronic chemical potentials, suggest that the corresponding 32CA reaction will have a non-polar character, which is in clear agreement with the analysis of the GEDT computed at the most favorable TS (see later). Note that although both reagents have a strong electrophilic character, their moderate nucleophilic character does not favour the GEDT.

**Figure 2:**
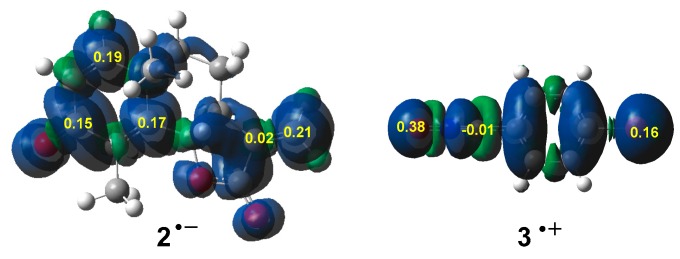
Three-dimensional representation of the Mulliken atomic spin densities (MASDs) (isovalue = 0.0004) of radical anion **2^−^** and radical cation **3**^+^, together with the electrophilic $P_{k}^{+}$ Parr functions of α-santonin derivative **2** and the nucleophilic $P_{k}^{-}$ Parr functions of nitrile oxide **3**. Blue regions correspond to positive values of the MASD, while green regions correspond to negative values of the MASD.

Although this 32CA reaction will present a non-polar character, in 2004, Domingo showed that the electrophilic ethylene derivative participating in 32CA reactions controls the asynchronicity in the bond formation process, regardless of the polar character of the reaction [44]. Thus, the formation of the first new single bond always involves the most electrophilic center of the ethylene derivative. Therefore, the electrophilic $P_{k}^{+}$ Parr functions [32] of *α*-santonin derivative **2** were analyzed (see Figure 2). The nucleophilic $P_{k}^{-}$ Parr functions of nitrile oxide **3** were also computed.

Figure 2 shows that the Mulliken atomic spin densities (MASDs) of the radical anion of *α*-santonin derivative **2** are mainly gathered at the β-conjugated positions of the three C–C double bonds of this compound. For the most reactive C4–C5 double bond, the electrophilic $P_{k}^{+}$ Parr functions of the C4 and C5 carbon centers, 0.21 and 0.02, respectively, indicate that the first single bond formation will involve the C4 carbon. Finally, the O3 oxygen of nitrile oxide **3** presents the most nucleophilic activation, $P_{k}^{-}$ = 0.38, whereas the C1 carbon is slightly nucleophilically deactivated.

### 3.3. Study of the Energy Profile Associated with the 32CA Reaction between α-Santonin Derivative **2** and p-Bromophenyl Nitrile Oxide **3**

**Scheme 3:**
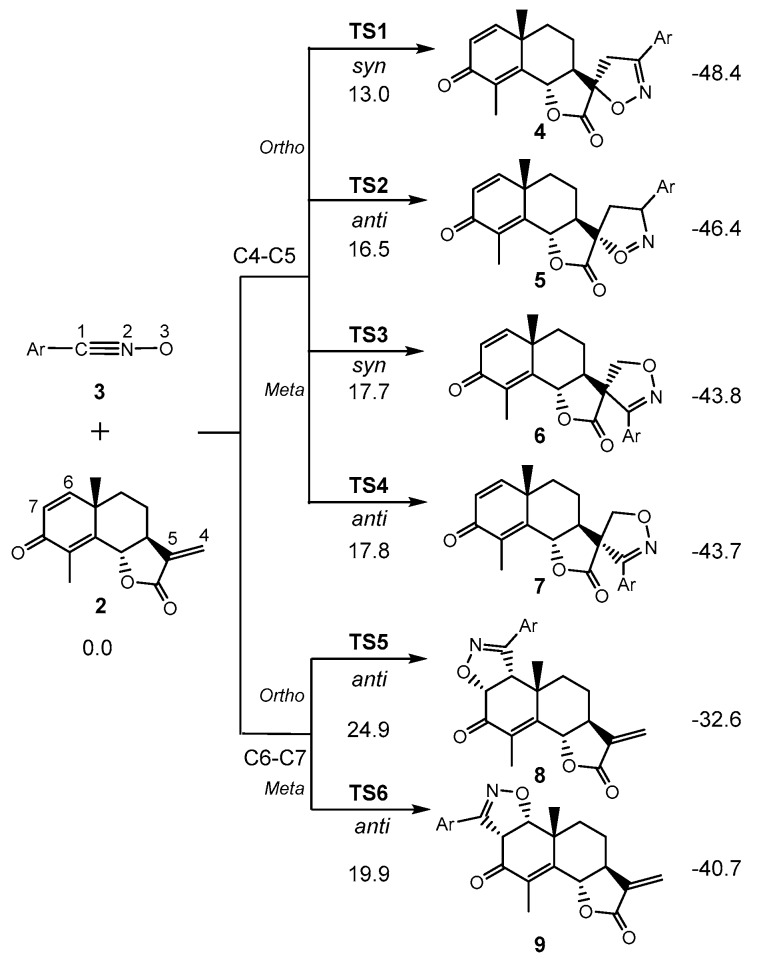
Studied reaction paths of the 32CA reaction of α-santonin derivative **2** with *p*-bromophenyl nitrile oxide **3**. MPWB1K/6-311G(d,p) relative energies in tetrahydrofuran (THF) are in kcal·mol^−1^.

Due to the presence of three C–C and two C–O double bonds in α-santonin derivative **2**, and the non-symmetry of both reagents, up to 20 reaction paths may exist. The two regioisomeric reaction paths, labeled *ortho* and *meta,* and the two diastereofacial isomeric reaction paths, labeled *anti* and *syn*, associated with the participation of the exocyclic C4–C5 double bond of α-santonin derivative **2**, as well as the two *anti/ortho* and *anti/meta* regioisomeric reaction paths associated with the participation of the C6–C7 double bond, have been considered here (see Scheme 3). The *ortho* regioisomeric reaction paths are associated with the formation of the C1–C4 or C1–C6 single bonds, while the *meta* ones are associated with the formation of the O3–C4 or O3–C6 single bonds. On the other hand, the *syn* diastereofacial isomeric reaction paths are associated with the approach of *p*-bromophenyl nitrile oxide **3** to the C4–C5 double bond by the face of the angular methyl group of α-santonin derivative **2**, while the *anti* diastereofacial isomeric reaction paths are associated with the approach by the opposite face.

The search for stationary points along the six reaction paths allowed locating and characterizing the reagents, i.e., **2** and **3**, six TSs, i.e., **TS1**, **TS2**, **TS3**, **TS4**, **TS5**, and **TS6**, and the corresponding cycloadducts, i.e., **4**–**9**. Consequently, this 32CA reaction takes place through a one-step mechanism. The relative energies, in THF, are given in Scheme 3, while the total electronic energies in the gas phase and in THF are given in Table S1 in the Supplementary Material.

The activation energies range from 13.0 (**TS1**) to 24.9 (**TS5**), with the 32CA reaction being strongly exothermic from 32.6 (**8**) to 48.4 (**4**) kcal·mol^−1^ (see Scheme 3). Some appealing conclusions can be drawn from the gas phase energies. (i) The most favorable reaction path is associated with the *ortho/syn* approach mode, yielding the experimental spiroisoxazoline **4** via **TS1**. The activation energy associated with **TS1**, 13.0 kcal·mol^−1^, is very close to that associated with the non-polar 32CA reaction of benzonitrile oxide **9** with methyl acrylate **10**, 12.3 kcal·mol^−1^ (see Scheme 2). (ii) This 32CA reaction is completely regioselective, as **TS3** is 4.7 kcal·mol^−1^ higher in energy than **TS1**. (iii) This 32CA reaction is completely diastereofacial selective, since **TS2** is 3.5 kcal·mol^−1^ above **TS1**. (iv) This reaction is completely chemoselective, as **TS6** is 6.9 kcal·mol^−1^ above **TS1**. (v) The formation of spiroisoxazoline **4** is strongly exothermic: −48.4 kcal·mol^−1^. Consequently, this 32CA can be considered irreversible.

**Figure 3:**
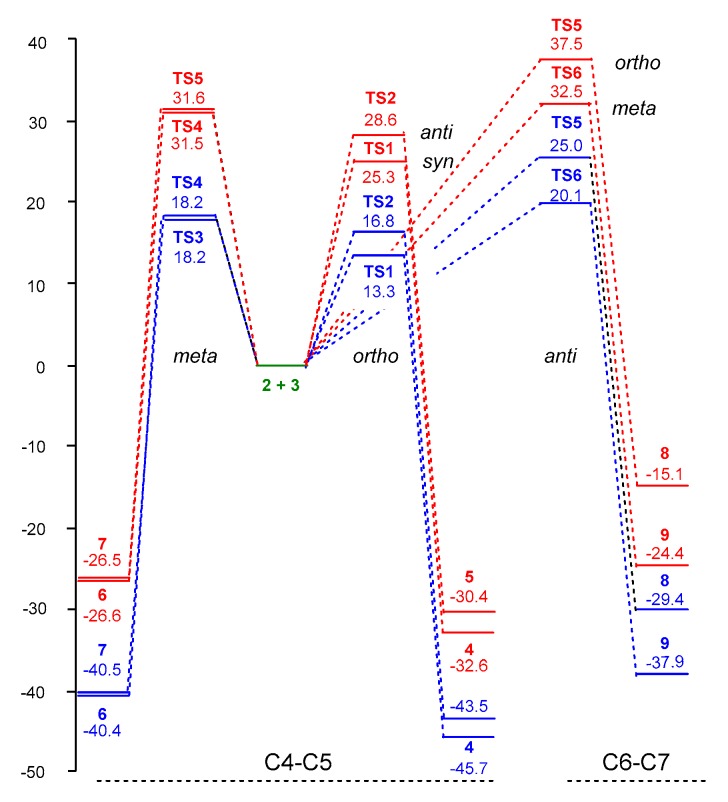
Enthalpy profiles, ΔH in blue (kcal·mol^−1^), and Gibbs free energy profiles, ΔG in red (kcal·mol^−1^), for the studied reaction paths of the 32CA reaction of α-santonin derivative **2** with *p*-bromophenyl nitrile oxide **3**, in the presence of THF at 0 °C.

The relative enthalpies and Gibbs free energy values for the stationary points involved in the studied reaction paths of the 32CA reaction of α-santonin derivative **2** with *p*-bromophenyl nitrile oxide **3** are given in Figure 3. The thermodynamic data are given in the Supplementary Material. The inclusion of thermal corrections to the electronic energies in THF does not substantially modify the relative enthalpies; while the activation enthalpies slightly increase by 0.1–0.5 kcal·mol^−1^, the reaction enthalpies slightly decrease by 2.8–3.3 kcal·mol^−1^. The inclusion of entropies to enthalpies strongly increases the activation Gibbs free energies by between 11.8–13.4 kcal·mol^−1^, and strongly decreases the reaction Gibbs free energies by between 13.0–14.0 kcal·mol^−1^, due to the unfavorable entropies associated with these bimolecular processes. The activation Gibbs free energy associated with the formation of spiroisoxazoline **4** becomes 25.3 kcal·mol^−1^, as the 32CA reaction is exergonic by 32.6 kcal·mol^−1^. The relative activation Gibbs free energies associated with the six TSs account for the complete C4–C5 chemoselectivity, *ortho* regioselectivity, and *syn* diastereofacial selectivity experimentally observed [10].

**Figure 4:**
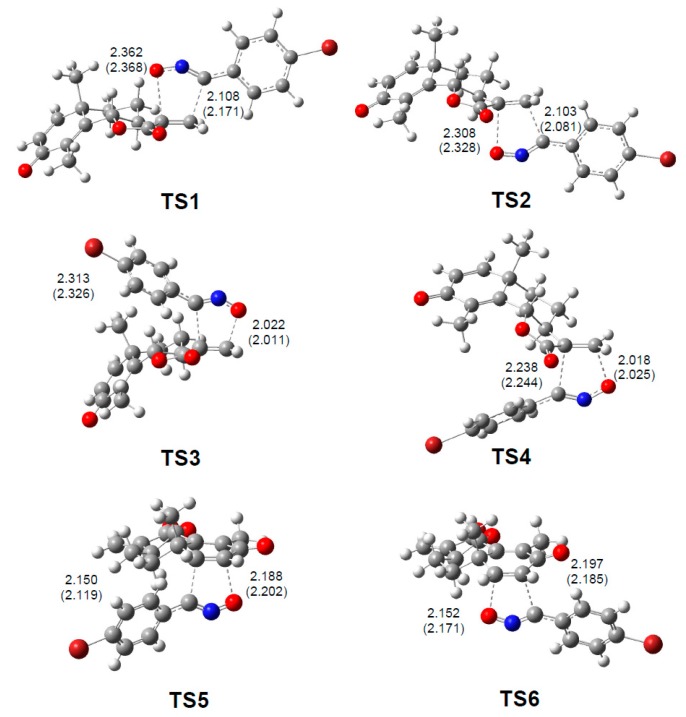
MPWB1K/6-311G(d,p) optimized geometries of the regioisomeric TSs involved in the 32CA reaction of α-santonin derivative **2** with *p*-bromophenyl nitrile oxide **3**. Values in THF are given in parentheses. Distances are given in angstroms, Å.

The gas phase geometries of the six TSs are given in Figure 4. At the *ortho* TSs associated with the attack to the C4–C5, the lengths of the C1–C4 and O3–C5 forming bonds are 2.108 Å and 2.362 Å at **TS1** and 2.103 Å and 2.308 Å at **TS2**, while at the *meta* TSs, the lengths of the C1–C5 and O3–C5 forming bonds are 2.022 Å and 2.313 Å at **TS3**, and 2.018 Å and 2.238 Å at **TS4**, respectively. At the *ortho* **TS5** associated with the attack to the C6–C7, the lengths of the C1–C6 and O3–C7 forming bonds are 2.150 Å and 2.188 Å, while at the *meta* **TS6**, the lengths of the C1–C5 and O3–C5 forming bonds are 2.197 Å and 2.152 Å, respectively.

The geometrical parameters of the TSs associated with the participation of the C4–C5 double bond are very similar to those found in the regioisomeric TSs associated with the non-polar *zw-type* 32CA reaction between benzonitrile oxide **9** and methyl acrylate **10** (see Scheme 2) [13]. Some appealing conclusions can be drawn from these geometrical parameters. (i) Considering that the formation of the C–C and C–O single bonds begins at the distance ranges of 2.0–1.9 Å [27] and 1.7–1.6 Å [12], respectively, these distances indicate that the formation of the new C–C and C–O single bonds has not yet begun in any of the six TSs (see later). (ii) At the six TSs, the shorter distance corresponds to the formation of the C–C(O) single bond involving the C4(C6) carbons of *α*-santonin derivative **2** [45]. (iii) The more favorable *ortho* TSs are slightly more asynchronous than the *meta* ones. (iv) The TSs involving the C6–C7 double bond of *α*-santonin derivative **2** are less asynchronous than those involving the C4–C5 double bond. (v) Inclusion of the solvent effect of THF scarcely modifies the gas phase geometries. In THF, the most favorable *ortho*/*syn* **TS1** is somewhat more delayed.

Finally, in order to evaluate the polar nature of the 32CA reaction of *α*-santonin derivative **2** with *p*-bromophenyl nitrile oxide **3**, the GEDT at the gas phase TSs was analyzed. Reactions with GEDT values of 0.0 e correspond to non-polar processes, while values higher than 0.2 e correspond to polar processes. The GEDT values computed at the TSs are: 0.02 e at **TS1**, 0.05 e at **TS2**, 0.02 e at **TS3**, 0.05 e at **TS4**, 0.02 e at **TS5** and 0.01 e at **TS6**. These very low values emphasize the non-polar character of this *zw-type* 32CA reaction, which is in clear agreement with the analysis of the CDFT indices at the ground state of the reagents (see Section 3.2), and account for the computed activation enthalpy via **TS1**, 13.3 kcal·mol^−^^1^ [13].

### 3.4. Origin of the Ortho Regioselectivity along the 32CA Reaction of α-Santonin Derivative **2** with p-Bromophenyl Nitrile Oxide **3**

Unlike the 32CA reaction of benzonitrile oxide **9** with methyl acrylate **10** (see Scheme 2), the 32CA reaction with α-santonin derivative **2** is completely *ortho* regioselective, as the *meta* **TS3** is 3.7 kcal·mol^−1^ higher in energy than the *ortho* **TS1**. While polar cycloaddition reactions are completely regioselective due to the favorable two-center interaction taking place between the most nucleophilic and the most electrophilic centers of both reagents, non-polar cycloaddition reactions are usually hardly regioselective [16]. Regioselectivity is mainly controlled by electronic factors associated with bonding changes along the reaction, although sometimes, stereoelectronic factors can control the regioselectivity [45]. The regioselectivity in the *zw-type* 32CA reactions of nitrones with substituted ethylenes was recently studied [16]. While polar 32CA reactions of nitrones with electrophilic ethylenes are *meta* regioselective, a change to the *ortho* regioselectivity is found in non-polar processes. In these cases, the most favorable reaction path corresponded to the earlier C–C bond formation involving the least nucleophilic carbon of nitrone [16].

In order to understand the origin of the complete *ortho* regioselectivity of this non-polar 32CA reaction, the 32CA reaction of the simplest nitrile oxide **13** with methyl acrylate **10** and with the simplest 3-methylene lactone **16** was analyzed. The energy and geometrical results are given in the Supplementary Material. Interestingly, the 32CA reaction between the simplest nitrile oxide **13** and 3-methylene lactone **16**, which does not present any stereoelectronic interaction, has a similar activation energy, 13.1 kcal·mol^−^^1^, and total *ortho* regioselectivity, ΔΔE_(ortho-meta)_ = 5.8 kcal·mol^−^^1^, to those associated with the 32CA reaction between α-santonin derivative **2** and *p*-bromophenyl nitrile oxide **3**. Consequently, the stereoelectronic factors associated with the approach of the two reagents do not control the regioselectivity of these 32CA reactions; the more favorable electronic changes along the C1–C4 than the C4–O3 bond formation are responsible for the *ortho* regioselectivity in these non-polar *zw-type* 32CA reactions.

### 3.5. Origin of the syn Diastereofacial Selectivity along the 32CA Reaction of α-Santonin Derivative **2** with p-Bromophenyl Nitrile Oxide **3**

**Figure 5:**
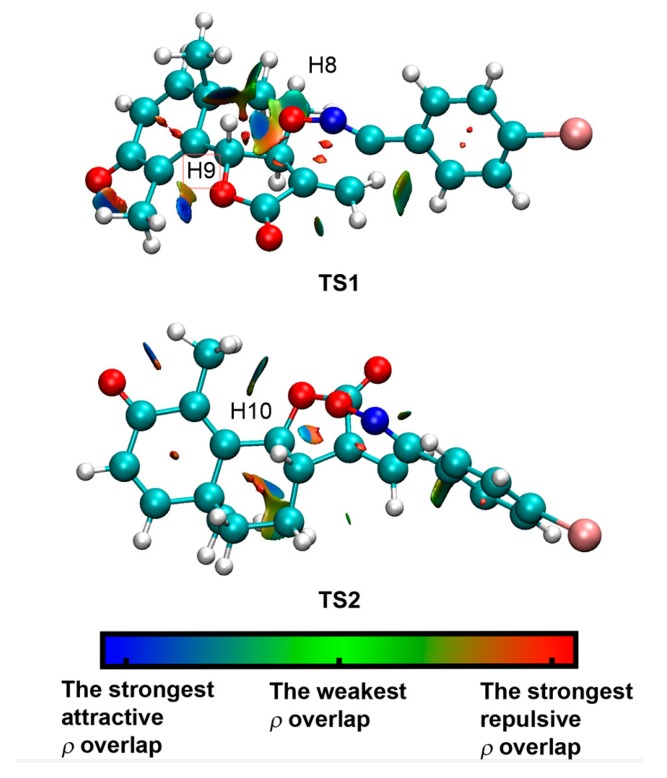
Non-covalent interactions (NCI) gradient isosurfaces of the TSs involved in the *ortho syn/anti* diastereroisomeric reaction paths associated with the 32CA reactions between α-santonin derivative **2** and *p*-bromophenyl nitrile oxide **3**. Surfaces are colored in the [−0.02, 0.02] a.u. range of sign(λ^2^)ρ (isosurfaces = 0.5 a.u.).

As has been commented, experimental evidence reveals that the 32CA reactions of *α*-santonin derivative **2** with *p*-bromophenyl nitrile oxide **3** are completely *ortho* regioselective and completely *syn* diastereofacial selective. In general, NCIs taking place at the TSs of 32CA reactions involving chiral species may be responsible for the diastereofacial selectivity [46]. Thus, an NCI topological analysis [36] of the electron density of the *ortho* regioisomeric pairs of *syn/anti* TSs associated with the 32CA reaction involving *α*-santonin derivative **2** and *p*-bromophenyl nitrile oxide **3** was performed. The corresponding NCI gradient isosurfaces are shown in Figure 5.

NCI topological analysis of the TSs shows the presence of a turquoise and a blue surface between the O3 oxygen of *p*-bromophenyl nitrile oxide **3** and the two H8 and H9 axial hydrogens, respectively, of α-santonin derivative **2** at **TS1**. Otherwise, at **TS2**, one of these surfaces is absent, while the other one involving the H10 axial hydrogen of the dihydrofuranone ring is also blue, in part, but smaller than that found at **TS1** (see Figure 5). In the NCI approach, the more overlapping electron density, the color of the corresponding NCI gradient isosurfaces shifts toward blue or red depending on the attractive or repulsive character of this overlapping, respectively [36]. However, it is worth emphasizing that NCI plots do not allow a quantitative analysis of the strength of the interactions. Consequently, this NCI analysis only allows revealing the presence of attractive overlaps between the electron densities belonging to the O3 and the three H8, H9, and H10 hydrogens; those related to the O3–H9 and O3–H10 interactions present higher amounts of electron density as shown by the darker blue color of the corresponding NCI surfaces. Anyway, at first sight, the presence of one more O3–H favorable interaction at **TS1** than at **TS2** might account for the higher stability of **TS1** than **TS2** by 4.0 kcal·mol^−1^ and, thus, for the experimental *syn* diastereofacial selectivity.

In order to quantify the nature of these non-covalent O3–H interactions, an IQA energy decomposition analysis [38,47,48] was performed. Due to molecular size restrictions, the IQA analysis was performed at reduced models of **TS1** and **TS2** (see **TS1r** and **TS2r** in Figure S3 in Supplementary Material), whose geometries were not optimized in order to keep the core lactone and nitrile oxide frameworks unvaried. As can be seen, an NCI topological analysis of smaller **TS1r** and **TS2r** confirmed very similar O3–H interaction patterns to those of the experimental **TS1** and **TS2** (see Figure S3 in the Supplementary Material). The total interaction energies (E_int_) and their classical (E_cl_) and exchange–correlation (E_xc_) contributions are given in Table 2.

The energy partitioning within the IQA scheme indicates that the three O3–H8, O3–H9, and O3–H10 interactions are favorable by 4.4 kcal·mol^−1^, 8.1 kcal·mol^−1^, and 5.7 kcal·mol^−1^, respectively; the O3–H9 interaction is the strongest one, which is in agreement with the somewhat shorter distance between the O3 and H9 centers. In the three cases, E_xc_, which is related to covalency, has a greater weight to E_int_ than E_cl_, which is associated with electrostatic forces; meanwhile, E_xc_ and E_cl_ contribute in relationships of ca. 60:40%, respectively, suggesting that the nature of these NCIs is partly covalent and electrostatic, with the covalent character being slightly more notable. Consequently, the strength of these O3–H interactions is mainly determined by their covalent nature, and therefore, the present IQA analysis is in agreement with the NCI analysis and allows concluding that the stronger O3–H9 interaction at **TS1** than the O3–H10 one at **TS2** could be responsible for the experimental *syn* diastereofacial selectivity.

### 3.6. ELF Topological Analysis of the C–C and C–O Bond Formation along the 32CA Reaction of α-Santonin Derivative **2** with p-Bromophenyl Nitrile Oxide **3**

In order to characterise the C–C and C–O bond formation along the 32CA reaction of α-santonin derivative **2** with *p*-bromophenyl nitrile oxide **3**, a topological analysis of the ELF along the IRC associated with the most favorable *ortho/syn* reaction path was performed, with the aim of finding the structures directly involved in the bond formation processes. The complete ELF analysis is given in Section 2 of the Supplementary Material.

Some appealing conclusions can be drawn from this ELF topological analysis. (i) The gas phase activation energy associated with the 32CA reaction between α-santonin derivative **2** and *p*-bromophenyl nitrile oxide **3** via **TS1**, 11.5 kcal·mol^−1^, can mainly be related to the rupture of the C1–N2 triple bond of the nitrile oxide **3**, which is in agreement with a *zw-type* mechanism [12]. Due to the non-polar character of this *zw-type* 32CA reaction, the corresponding bonding changes demand an energy cost of 16.4 kcal·mol^−1^ from the first structure of the reaction path. (ii) At **TS1**, neither the C1–C4 nor the O3–C5 single bonds have been yet formed, thus precluding any cyclic conjugation associated with a hypothetical aromatic TS (see Figure S2). (iii) The formation of the first C1–C4 single bond takes place at a C–C distance of ca. 1.96 Å by the C-to-C coupling of two C1 and C4 *pseudoradical* centers generated along the reaction path from the previous rupture of the corresponding multiple bonds [27] (see structures **S2** and **S3** in Figure S2). (iv) Formation of the second O3–C5 single bond takes place at an O–C distance of ca. 1.86 Å, by sharing the non-bonding electron density of the O3 oxygen and the C5 *pseudoradical* center (see structures **S4** and **S5** in Figure S2). (v) Taking into account the IRC values of the structures at which the formation of the two single bonds occurs, i.e., **S3** and **S5**, the bond formation can be considered highly asynchronous, despite the non-polar nature of the reaction. (vi) The sequential bonding changes that are demanded for the formation of the new single bonds clearly rules out the pericyclic mechanism for this 32CA reaction.

## 4. Conclusions

The 32CA reaction of α-santonin derivative **2**, having an exocyclic C–C double bond, with *p*-bromophenyl nitrile oxide **3**, yielding only a spiro-isoxazoline **4**, as experimentally carried out by Kumar [10], has been studied within the MEDT at the MPWB1K/6-311G(d,p) computational level.

Analysis of the CDFT reactivity indices at the ground state of the reagents suggests that this 32CA reaction will have a non-polar character due to the relatively low electrophilic and nucleophilic character of both reagents, which is a behavior confirmed by the analysis of the GEDT computed at the most favorable *ortho/syn* **TS1**.

This non-polar *zw-type* 32CA reaction presents an activation enthalpy of 13.3 kcal·mol^−1^. Analysis of the activation Gibbs free energies indicates that this 32CA reaction is completely chemoselective, *ortho* regioselective and *syn* diastereofacial selective, yielding only spiroisoxazoline **4** under kinetic control, in complete agreement with the experimental outcomes.

The more favorable C–C bond formation involving the β-conjugated carbon of the α-santonin derivative and the carbon of nitrile oxide than the C–O bond formation involving the oxygen of nitrile oxide in this non-polar 32CA reaction, rather than the hindrance along the *meta* approach modes, is responsible for the total *ortho* regioselectivity. On the other hand, NCI and IQA analyses along the two more favorable *ortho* TSs indicate that the favorable electronic interactions taking place between the oxygen of the nitrile oxide and two axial hydrogen atoms of the α-santonin derivative are responsible for the total *syn* diastereofacial selectivity that was experimentally found.

This MEDT study emphasizes how the analysis of electron density along a chemical reaction allows achieving a complete comprehension of organic reactivity.

## Figures and Tables

**Table 1:** B3LYP/6-31G(d) electronic chemical potential (μ), chemical hardness (η), electrophilicity (ω), and nucleophilicity (*N*), in eV, of α-santonin derivative **2** and *p*-bromophenyl nitrile oxide **3**, nitrile oxide **13** and 3-methylene lactone **16**.

	μ	η	ω	*N*
α-santonin derivative **2**	−4.16	4.90	1.76	2.51
*p*-bromophenyl nitrile oxide **3**	−4.03	4.81	1.69	2.69
3-methylene lactone **16**	−4.29	5.99	1.54	1.84
simplest nitrile oxide **13**	−3.40	7.94	0.73	1.75

**Table 2:** B3LYP/6-31G(d) Total interaction energy *(E_int_)*, classical energy *(E_cl_)* and exchange–correlation energy *(E_xc_)*, in kcal·mol^−1^, for the O3–H8, O3–H9, and O3–H10 interactions of small models **TS1r** and **TS2r**.

	*E_int_*	*E_cl_*	*E_xc_*
O3–H8	−4.4	−1.6	−2.8
O3–H9	−8.1	−3.1	−5.0
O3–H10	−5.7	−2.0	−3.6

## Supplementary Materials

The following are available online. 32CA reactions of simplest nitrile oxide **13** with methyl acrylate **10** and with 3-methylene lactone **16**. ELF topological analysis of C–C and C–O bond formation along the 32CA reaction of α-santonin derivative **2** with *p*-bromophenyl nitrile oxide **3**. Tables with the MPWB1K/6-311G(d,p) total and relative energies, in gas phase and in THF, and with total and relative enthalpies, entropies, and Gibbs free energies for the stationary points involved in the 32CA reaction of α-santonin derivative **2** with *p*-bromophenyl nitrile oxide **3**. Figure with the NCI gradient isosurfaces of **TS1r** and **TS2r**. MPWB1K/6-311G(d,p) Cartesian coordinates, in THF, of the stationary points involved in the 32CA reactions of α-santonin derivative **2** with *p*-bromophenyl nitrile oxide **3**.
