# Supplementary material for: A Molecular Electron Density Theory Study of the Chemoselectivity, Regioselectivity, and Diastereofacial Selectivity in the Synthesis of an Anticancer Spiroisoxazoline derived from α-Santonin
Source: Molecules. 2019 Feb 26;24(5):832. doi: 10.3390/molecules24050832 (PMC6429143; doi:10.3390/molecules24050832)
Supplement: Supplementary file 1 [file molecules-24-00832-s001.pdf]

Supplementary Material

A Molecular Electron Density Theory Study of the Chemoselectivity, Regioselectivity, and Diastereofacial Selectivity in the Synthesis of an Anticancer SpiroIsoxazoline derived from α-Santonin

Luis R Domingo [1,*] Mar Ríos-Gutiérrez[1] and Nivedita Acharjee [2,*]

[1]*Department of Organic Chemistry, University of Valencia, Dr. Moliner 50, E-46100 Burjassot, Valencia, Spain*

[2]*Department of Chemistry, Durgapur Government College, J. N. Avenue, Durgapur-713214, West Bengal, India, E-mail: nivchem@gmail.com; Tel. No: 09474150273*

Index

S2 32CA reactions of simplest nitrile oxide **13** with methyl acrylate **10** and 3-methylene lactone **16**

S5 ELF topological analysis of C‑C and C‑O bond formation along the 32CA reaction of α-santonin derivative **2** with p-bromophenyl nitrile oxide **3**

S9 Figure with the non-covalent interactions (NCI) gradient isosurfaces of **TS1r** and **TS2r**

S10 Table with the MPWB1K/6-311G(d,p) total and relative energies, in gas phase and in THF, of the stationary points involved in the 32CA reaction between *α*-santonin derivative **2** and *p*-bromophenyl nitrile oxide **3**

S11 Table with the MPWB1K/6-311G(d,p) total and relative enthalpies, entropies, and Gibbs free energies, computed at 0 ºC and one atm in THF, for the stationary points involved in the 32CA reaction of *α*-santonin derivative **2** with *p*-bromophenyl nitrile oxide **3**

S12 MPWB1K/6-311G(d,p) Cartesian coordinates, in THF, of the stationary points involved in the 32CA reactions of *α*-santonin derivative **2** with *p*-bromophenyl nitrile oxide **3**

*1. 32CA reactions of the simplest nitrile oxide **13** with methyl acrylate **10** and 3-methylene lactone **16***

In order to characterize the origin of the total regioselectivity found in the 32CA reaction of α-santonin derivative **2** with p-bromophenyl nitrile oxide **3**, the 32CA reactions of the simplest nitrile oxide **13** with methyl acrylate **10** and 3-methylene lactone **16** were studied in gas phase at the MPWB1K/6-311G(d,p) computational level (see Scheme S1). For the 32CA reaction between the simplest nitrile oxide **13** and methyl acrylate **10**, the *meta* **TS8** was found to be 1.4 kcal·mol^{-1} higher in energy than the *ortho* **TS7**, indicating that this non-polar *zw-type* 32CA reaction is poorly regioselective. A similar poor regioselectivity was found for the 32CA reaction between benzonitrile oxide **9** and methyl acrylate **10** at the B3LYP/6-31G(d) (see Scheme 2).

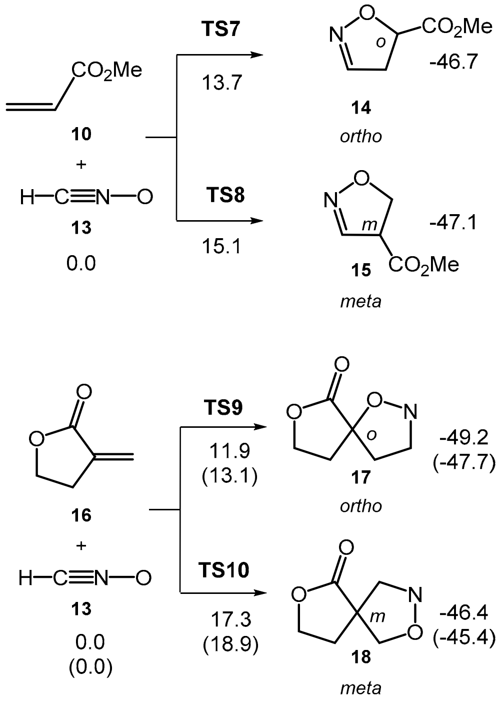

Scheme S1. 32CA reactions of the simplest nitrile oxide **13** with methyl acrylate **10** and 3-methylene lactone **16**. Relative MPWB1K/6-311G(d,p) gas phase energies are given in kcal·mol^{-1}. Relieve energies in THF are given in parentheses.

Interestingly, when the 32CA reaction of the simplest nitrile oxide **13** with the simplest 3-methylene lactone **16** is analyzed, while the activation energy associated with the *ortho* **TS9** decreases to 11.9 kcal·mol^{-1}, the energy difference with the *meta* **TS10** increases to 5.4 kcal·mol^{-1}, which is in clear agreement with the energy results found in the 32CA reaction of α-santonin derivative **2** with p-bromophenyl nitrile oxide **3** (see Scheme 3). Considering that only minor steric factors exist along the approach of the

simplest nitrile oxide **13** to the exocyclic C–C double bond of lactone **16**, the total regioselectivity found in this 32CA reaction can be exclusively associated with electronic factors related to the bonding changes along the reaction.

The inclusion of solvent effects of THF increases the activation energies by between 1.2–1.6 kcal·mol^{-1}, and decreases the exothermic character of the reaction by between 1.0–1.5 kcal·mol^{-1} as a consequence of a better solvation of the reagents than TSs and cycloadducts.

The geometries of the *ortho* and *meta* regioisomeric TSs associated with the 32CA reaction between the simplest nitrile oxide **13** and 3-methylene lactone **16** are given in Figure S1. A comparison of the distances between the nuclei involved in the formation of the new C–C and C–O single bonds at these TSs with those involved in the 32CA reaction of α-santonin derivative **2** with *p*-bromophenyl nitrile oxide **3** (see Figure S1) indicates that while the more favorable *ortho* TSs show a very similar bond formation pattern, the *meta* TSs involving *p*-bromophenyl nitrile oxide **3** are slightly more asynchronous as a consequence of the presence of the phenyl substituent in nitrile oxide **3**. Inclusion of the solvent effects of THF scarcely modifies the gas phase geometries.

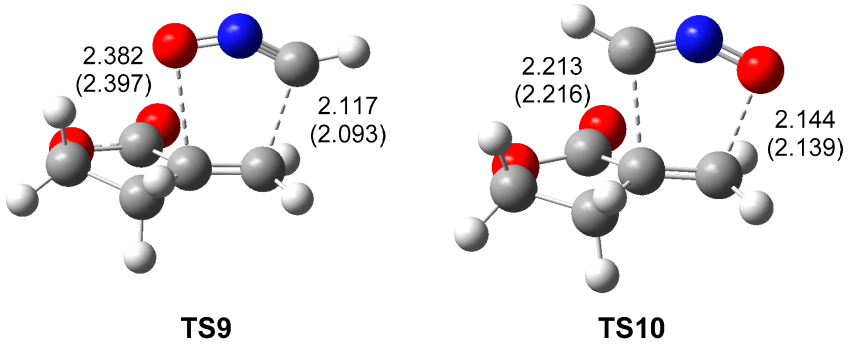

Figure S1. MPWB1K/6-311G(d,p) gas phase optimized geometries of the regioisomeric transition states (TSs) involved in the 32CA reaction of the simplest nitrile oxide **13** with 3-methylene lactone **16**. Values in THF are given in parentheses. Distances are given in angstroms, Å.

Finally, in order to evaluate the polar nature of the 32CA reaction of the simplest nitrile oxide **13** with 3-methylene lactone **16**, the GEDT at the gas phase TSs was analyzed. The computed GEDT values, 0.05 e at **TS9** and 0.00 e at **TS10**, emphasize the non-polar character of this *zw-type* 32CA reaction. In spite of the electrophilic character

of simplest 3-methylene lactone **16**, ω = 1.54 eV, the very low nucleophilic character of simplest nitrile oxide **13**, *N* = 1.17 eV, accounts for the non-polar character of this 32CA reaction (the global reactivity indices of nitrile oxide **13** and 3-methylene lactone **16** are given in Table 1).

*2. ELF topological analysis of C–C and C–O bond formation along the 32CA reaction of α-santonin derivative **2** with p-bromophenyl nitrile oxide **3***

In order to characterize the C–C and C–O bond formation along the 32CA reaction of *α*-santonin derivative **2** with *p*-bromophenyl nitrile oxide **3**, a topological analysis of the ELF along the IRC associated with the most favorable *ortho/syn* reaction path was performed, with the aim of finding the structures directly involved in the bond formation processes. The populations of the most relevant ELF valence basins, GEDT, relative energies, C–C and C–O forming bond distances, and IRC values of the selected structures of the IRC are gathered in Table S1, while the ELF valence basin attractor positions are represented in Figure S2.

The *ortho/syn* reaction path begins at structure **S1**, d(C1−C4) = 3.480 Å and d(O3−C5) = 3.241 Å. Thus, it is expected that its ELF topological features will be very similar to those of the separated reagents. While the two V(C1,N2) and V′(C1,N2) disynaptic basins of the nitrile oxide framework and the two V(C4,C5) and V′(C4,C5) disynaptic basins of the santonin exocyclic ethylene moiety have total populations of 5.97e and 3.43e, respectively, the V(N2,O3) disynaptic basin and the V(O3) monosynaptic basin integrate 1.84e and 5.69e. Therefore, these populations show a similar bonding pattern to that of the separated reagents; i.e. a C1−N2 triple bond, an N2−O3 single bond, an O3 oxygen non-bonding electron density equivalent to three lone pairs, and an underpopulated C4−C5 double bond (Section 3.1).

At **TS1**, d(C1−C4) = 2.130 Å and d(O3−C5) = 2.362 Å, together with the strong depopulation of the two V(C1,N2) and V′(C1,N2) disynaptic basins to 4.08e, which allows relating the C1−N2 bonding region to a double bond, a new V(N2) monosynaptic basin is observed with a population of 1.90e. This V(N2) monosynaptic basin can be related to the N2 non-bonding electron density present at the final spiroisoxazoline **4**. Parallel, the depopulation of the V(C1,C1’) disynaptic basin associated with the C1−C1’ bonding region (not shown in Table S2), has caused the appearance of a V(C1)

monosynaptic basin integrating 0.40e at **TS1**, which can be associated with a C1 *pseudoradical* center. On the other hand, at the santonin framework, the C4–C5 bonding region has been depopulated by 0.27e, in such a manner that the two V(C4,C5) and V′(C4,C5) disynaptic basins have merged into a single V(C4,C5) disynaptic basin integrating 3.16e. Otherwise, although the total population associated with the O3 oxygen has decreased by only 0.09e, the V(O3) monosynaptic basin has split into two V(O3) and V′(O3) monosynaptic basins, integrating 2.85e and 2.75e. At **TS2**, no disynaptic basin associated with the new C1−C4 or O3−C5 single bonds are found.

At structure **S2**, which is exactly before the formation of the first C1−C4 single bond, d(C1−C4) = 1.973 Å and d(O3−C5) = 2.265 Å, while the V(C1) monosynaptic basin reaches 0.66e, and a new V(C4) monosynaptic basin, integrating 0.37e, is observed at the ethylene system of the santonin framework (see Figure S2). This monosynaptic basin, which can also be related with a C4 *pseudoradical* center, entirely comes from the depopulation of the V(C4,C5) disynaptic basin to 2.80e. At **S2**, the two C1 and C4 *pseudoradical* centers demanded for the corresponding C1−C4 bond formation by sharing electron density are observed.

At structure **S3**, d(C1−C4) = 1.956 Å and d(O3−C5) = 2.254 Å, the two V(C1) and V(C4) monosynaptic basins merge into a new V(C1,C4) disynaptic basin integrating an initial population of 1.05e (see Figure S2). Thus, formation of the first C1−C4 single bond takes place at a C–C distance of ca. 1.96 Å by sharing the non-bonding electron density of two C1 and C4 *pseudoradical* centers.

At structure **S4**, d(C1−C4) = 1.597 Å and d(O3−C5) = 1.884 Å, a new V(C5) monosynaptic basin, integrating 0.25e, is observed as a consequence of the depopulation of the V(C4,C5) disynaptic basin to 2.19e, as well as the depopulation of the two adjacent V(C5,C5′) and V(C5,C5″) disynaptic basins (not shown in Table S1). Note that the depopulation of the V(C1,N2) and V(C4,C5) disynaptic basins by 0.42e and 0.59e, respectively, may have also contributed to the increase of the V(C1,C4) population to 1.85e. At **S4**, while the C1−N2 bonding region can be characterized as an underpopulated double bond, the population of the V(N2,O3) disynaptic basin, 1.27e, suggests an underpopulated N2−O3 single bond.

At structure **S5**, d(C1−C4) = 1.590 Å and d(O3−C5) = 1.865 Å; while the total population of the two V(O3) and V′(O3) monosynaptic basins decreases by 0.31e and the V(C5) monosynaptic basin disappears, a new V(O3,C5) disynaptic basin is created with

an initial population of 0.56e (see Figure S2). This relevant topological change indicates that the formation of the second O3–C5 single bond takes place at an O–C distance of ca. 1.86 Å, by sharing the non-bonding electron densities of the O3 oxygen and the C5 *pseudoradical* center. Interestingly, the extremely low population of the V(O3,C5) disynaptic basin, which is half that of the V(C1,C4) disynaptic basin at **S3**, suggests an incipient remarkably polarized O3–C5 single bond. Note that the population associated with the O3 oxygen is 5.42e.

Finally, at spiroisoxazoline **4**, d(C1–C4) = 1.494 Å and d(O3–C5) = 1.433 Å, as the total population of the two V(O3) and V′(O3) monosynaptic basin decreases to 4.96e, the population of the V(O3,C5) disynaptic basin strongly increases to 1.34e, but remains low compared to that of the V(C1,C4) disynaptic basin, which reaches 1.99e. Similarly, the V(N2,O3) disynaptic basin ends up integrating only 1.06e, while the V(N2) monosynaptic basin reaches 2.87e.

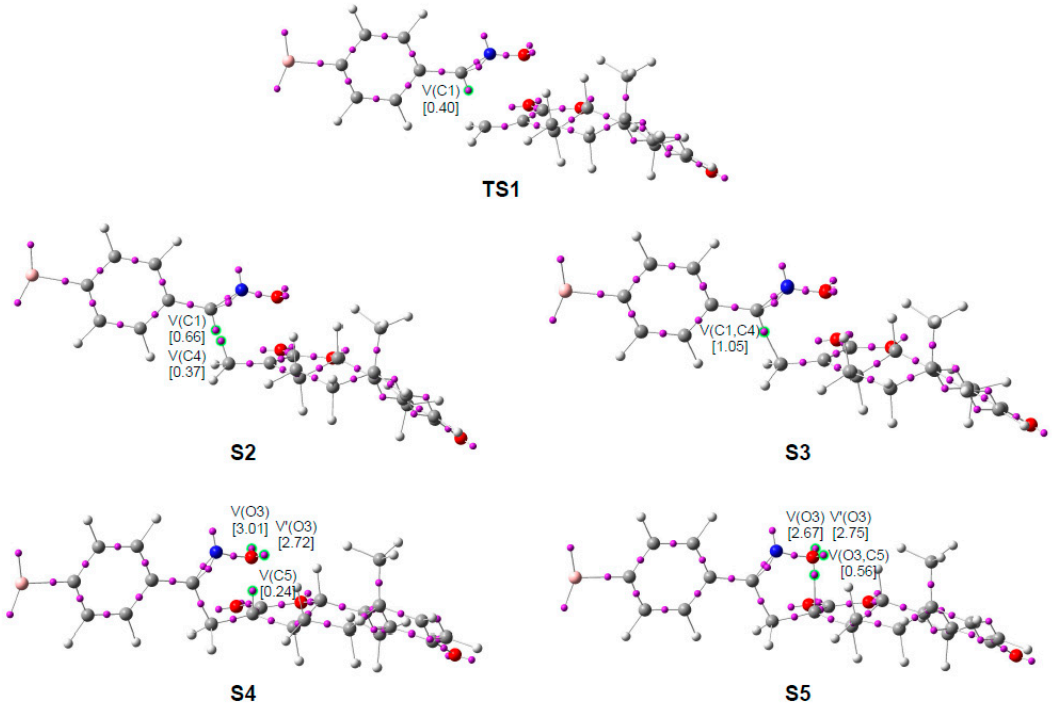

Figure S2. Attractor positions of the ELF valence basins for the selected structures associated with the formation of the C1–C4 and O3–C5 single bonds along the most favorable *ortho/syn* reaction path of the non-polar *zw-type* 32CA reaction between *α*-santonin derivative **2** and *p*-bromophenyl nitrile oxide **3**. The electron populations, in average number of electrons, e, are given in brackets.

Table S1. ELF valence basin populations, intrinsic reaction coordinate (IRC) values, internuclear distances along the single bond formation, relative[a] electronic energies and global electron density transfer (GEDT) of the selected structures associated with the formation of the C1–C4 and O3–C5 single bonds along the most favorable *ortho/syn* reaction path of the non-polar *zw-type* 32CA reaction between α-santonin derivative **2** and *p*-bromophenyl nitrile oxide **3**, yielding spiroisoxazoline **4**. IRC values are given in atomic unities, a.u., distances in angstroms, Å, GEDT values and electron populations are given in average number of electrons, e, and relative energies are given in kcal·mol^{-1}.

Structures	**S1**	**TS1**	**S2**	**S3**	**S4**	**S5**	**4**
IRC	-18.81	0.00	0.92	1.02	3.68	3.79	-
d(C1–C4)	3.480	2.130	1.973	1.956	1.597	1.590	1.494
d(O3–C5)	3.241	2.362	2.265	2.254	1.884	1.865	1.433
ΔE[a]	0.0	16.4	13.8	13.1	-17.1	-18.4	-45.4
GEDT	0.01	0.02	-0.04	-0.05	-0.23	-0.23	-0.28
V(C1,N2)	3.13	2.01	1.86	1.85	1.64	1.63	1.55
V'(C1,N2)	2.84	2.07	1.89	1.88	1.67	1.65	1.63
V(N2)		1.90	2.17	2.18	2.61	2.63	2.87
V(N2,O3)	1.84	1.62	1.56	1.55	1.27	1.25	1.06
V(C4,C5)	1.75	3.16	2.80	2.78	2.19	2.19	2.03
V'(C4,C5)	1.68						
V(C1)			0.66				
V(C4)			0.37				
V(O3)	5.69	2.85	2.85	2.91	3.01	2.67	2.43
V'(O3)		2.75	2.85	2.84	2.72	2.75	2.53
V(C5)					0.24		
V(C1,C4)				1.05	1.85	1.86	1.99
V(O3,C5)						0.56	1.34
V(C1,C1')	2.54	2.37	2.32	2.30	2.34	2.34	2.36
V(C5,C5')	2.31	2.37	2.42	2.44	2.31	2.32	2.22
V(C5,C5")	2.07	2.10	2.11	2.13	2.09	2.10	2.01

(a) Relative to the first structure of the reaction path, **S1**

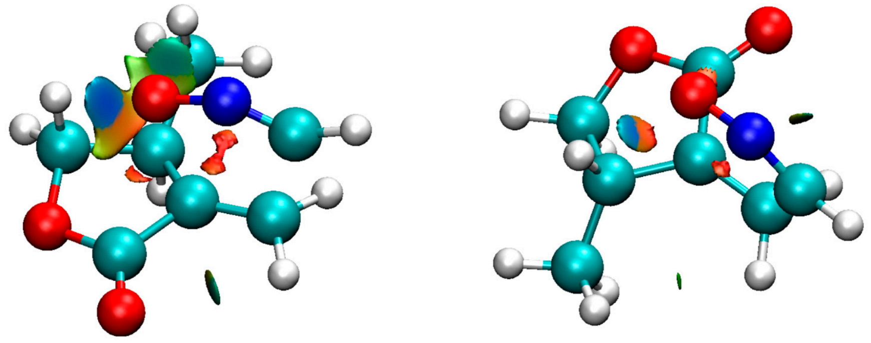

TS1r **TS2r**

Figure S3. NCI gradient isosurfaces of **TS1r** and **TS2r**, as reduced models for the interacting quantum atoms (IQA) calculations of **TS1**, and **TS2**, respectively. Surfaces are colored in the [–0.02, 0.02] a.u. range of sign(λ^2)ρ (isosurfaces = 0.5 a.u.).

Table S2. MPWB1K/6-311G(d,p) total (E, in a.u.) and relative[a] (ΔE, in kcal·mol^{-1}) energies, in gas phase and in THF, of the stationary points involved in the 32CA reaction between *α*-santonin derivative **2** and *p*-bromophenyl nitrile oxide **3**.

	E	ΔE	E	ΔE
	Gas phase		THF	
**2**	–806.675001		–806.687480	
**3**	–2973.408221		–2973.414530	
**TS1**	–3780.064931	11.5	–3780.081237	13.0
**TS2**	–3780.058533	15.5	–3780.075689	16.5
**TS3**	–3780.059044	15.2	–3780.073820	17.7
**TS4**	–3780.058621	15.4	–3780.073669	17.8
**TS5**	–3780.044552	24.3	–3780.062296	24.9
**TS6**	–3780.056884	16.5	–3780.070294	19.9
**4**	–3780.163551	–50.4	–3780.179211	–48.4
**5**	–3780.157904	–46.9	–3780.175899	–46.4
**6**	–3780.156908	–46.2	–3780.171748	–43.8
**7**	–3780.155677	–45.5	–3780.171699	–43.7
**8**	–3780.137387	–34.0	–3780.15394	–32.6
**9**	–3780.152016	–43.2	–3780.166797	–40.7

Table S3. MPWB1K/6-311G(d,p) enthalpies (H, in a.u.), entropies (S, in cal·mol^{-1}·K^{-1}), and Gibbs free energies (G, in a.u.); and relative[a] enthalpies (ΔH, in kcal·mol^{-1}), entropies (ΔS, in cal·mol^{-1}·K^{-1}) and Gibbs free energies (ΔG, in kcal·mol^{-1}), computed at 0 ºC and one atm in THF, for the stationary points involved in the 32CA reaction of *α*-santonin derivative **2** with *p*-bromophenyl nitrile oxide **3**.

	H	ΔH	S	ΔS	G	ΔG
**2**	–2973.310550		91.3		–2973.350304	
**3**	–806.386783		117.5		–806.437927	
**TS1**	–3779.676120	13.3	164.9	–43.9	–3779.747899	25.3
**TS2**	–3779.670558	16.8	165.5	–43.3	–3779.742586	28.6
**TS3**	–3779.668302	18.2	159.7	–49.1	–3779.737812	31.6
**TS4**	–3779.668374	18.2	160.1	–48.7	–3779.738074	31.5
**TS5**	–3779.657488	25.0	163.1	–45.8	–3779.728467	37.5
**TS6**	–3779.665332	20.1	163.5	–45.3	–3779.736507	32.5
**4**	–3779.770111	–45.7	161.1	–47.7	–3779.840228	–32.6
**5**	–3779.766702	–43.5	160.6	–48.2	–3779.836609	–30.4
**6**	–3779.761906	–40.5	157.8	–51.0	–3779.830589	–26.6
**7**	–3779.761728	–40.4	157.8	–51.0	–3779.830415	–26.5
**8**	–3779.744133	–29.4	156.5	–52.3	–3779.812248	–15.1
**9**	–3779.757680	–37.9	159.5	–49.3	–3779.827117	–24.4

*MPWB1K/6–311G(d,p) Cartesian coordinates, in THF, of the stationary points involved in the 32CA reactions of α-santonin derivative **2** with p-bromophenyl nitrile oxide **3**.*

2

C	2.87023600	1.01188400	–0.32922200
C	1.44003500	1.18890500	0.00468200
C	0.69016400	0.11094000	0.25935000
C	2.64466000	–1.39328200	–0.10695900
C	3.39964400	–0.34439700	–0.38750700
H	3.05858200	–2.39155300	–0.14290400
H	4.43745700	–0.43848400	–0.66211900
O	3.58792100	1.96182700	–0.56140400
C	0.96436000	2.60754600	0.00930600
H	1.82452900	3.25913900	–0.07179800
H	0.30523300	2.80794500	–0.82970000
H	0.41697500	2.84468900	0.91219000
C	0.42212100	–2.21808400	–0.66915400
H	0.66409800	–1.92664600	–1.68986600
H	0.77576500	–3.23777000	–0.53682600
C	–1.08542500	–2.14721000	–0.48927300
H	–1.57022700	–2.73426900	–1.26410300
H	–1.39432200	–2.56182400	0.46858500
C	–1.47971300	–0.70034200	–0.57635700
H	–1.13086300	–0.29698000	–1.53163500
C	–0.77538600	0.09883100	0.51061500
H	–0.96672800	–0.37923800	1.47114900
C	1.21947600	–1.30657300	0.29235500
C	1.13933600	–1.87030600	1.72073600
H	0.11605500	–2.01366400	2.05009500
H	1.63144400	–2.83818900	1.75478000
H	1.63747900	–1.20718700	2.42063700
O	–1.45915000	1.34403500	0.53111800
C	–2.87141100	–0.22778400	–0.37289300
C	–2.74022400	1.14545200	0.17202200
O	–3.57169300	1.98661200	0.29434900
C	–4.03757400	–0.81065300	–0.56679000
H	–4.94779100	–0.27941500	–0.33931300
H	–4.11610500	–1.81591500	–0.94713200

3

C	–3.09268200	–0.00028500	0.00000900
N	–4.23996600	–0.00009300	0.00000300
O	–5.43700300	0.00041900	–0.00000600
C	–1.67382500	–0.00019300	0.00000600
C	–0.97778300	1.20264900	0.00000400
C	–0.97763400	–1.20294800	0.00000400

C	0.39911100	1.20393200	0.00000100
H	–1.51590300	2.13473600	0.00000500
C	0.39926000	–1.20405900	0.00000100
H	–1.51563900	–2.13510200	0.00000500
C	1.07498300	–0.00002200	0.00000000
H	0.94062700	2.13340700	0.00000000
H	0.94089000	–2.13346700	0.00000000
Br	2.95477800	0.00009400	–0.00000400

TS1

C	6.51225600	–0.26049700	–0.60559900
C	5.31994400	0.51711100	–0.21061800
C	4.18499800	–0.12058900	0.09805300
C	5.28755300	–2.32760700	–0.31816700
C	6.40838800	–1.71166900	–0.65489100
H	5.22481100	–3.40668600	–0.34886300
H	7.28924800	–2.24619400	–0.97091300
O	7.54852100	0.30051900	–0.89517200
C	5.55301400	1.99071000	–0.22971100
H	6.48700100	2.20436600	0.28056800
H	5.68496300	2.33260100	–1.25400900
H	4.75357100	2.54976800	0.22577700
C	2.90745400	–2.11999500	–0.77380600
H	3.20781000	–1.96737400	–1.80916500
H	2.79734300	–3.19211900	–0.62901800
C	1.58371600	–1.41039800	–0.54345200
H	0.86341400	–1.73705900	–1.28837800
H	1.16891100	–1.63755600	0.43655700
C	1.84692200	0.06615400	–0.64275400
H	2.30007500	0.27598000	–1.61732300
C	2.85853700	0.47384400	0.41774800
H	2.51964000	0.07713200	1.37044700
C	4.05895700	–1.63238600	0.13677400
C	3.80881300	–2.10860700	1.57775300
H	2.83106500	–1.81568400	1.94465600
H	3.85876800	–3.19332000	1.61254700
H	4.56316500	–1.70892600	2.24787600
O	2.73498800	1.88507700	0.51031300
C	0.79559800	1.08837700	–0.42408700
C	1.47795300	2.24317600	0.18922300
O	1.06731400	3.34728400	0.36406500
C	–0.47640300	1.15970200	–0.88339300
H	–0.84358700	0.41838500	–1.57586300
H	–0.95513500	2.12736300	–0.87761400
O	0.31027200	0.41891000	1.79436800
C	–1.68252700	0.43593600	0.68695000
N	–0.86790500	0.31236900	1.54758800
C	–3.07450300	0.22700200	0.40567900

C	–3.67816600	0.82297800	–0.69090400
C	–3.82524700	–0.58906600	1.24651600
C	–5.01588800	0.61135500	–0.94778200
H	–3.11129100	1.45929700	–1.34738900
C	–5.16115300	–0.79911500	0.99566600
H	–3.35780900	–1.05469100	2.09706200
C	–5.74567100	–0.19740700	–0.10236000
H	–5.48387500	1.07571300	–1.79771600
H	–5.74225700	–1.42803800	1.64696700
Br	–7.56961500	–0.48822600	–0.44939800

TS2

C	–6.21539100	–0.00399700	–1.20521300
C	–5.11111200	–0.61765300	–0.43745100
C	–4.26215900	0.17161600	0.22954800
C	–5.46294100	2.20993800	–0.55411200
C	–6.30665200	1.45059000	–1.23282500
H	–5.55347300	3.28714100	–0.57865500
H	–7.10226000	1.86538200	–1.82985100
O	–7.02061100	–0.68062000	–1.80987800
C	–5.04559200	–2.11117400	–0.49974400
H	–5.91794100	–2.47341500	–1.02767100
H	–4.15851700	–2.44722400	–1.02677900
H	–5.01748400	–2.55234800	0.48867600
C	–3.05714800	2.34618000	–0.18098600
H	–2.97440900	2.19624000	–1.25609000
H	–3.14538300	3.41711300	–0.01412700
C	–1.79887400	1.80224000	0.47853200
H	–0.92669400	2.25326900	0.01326100
H	–1.76347400	2.05042200	1.53814300
C	–1.81058700	0.31014600	0.29132100
H	–1.89121000	0.09222300	–0.77412600
C	–3.04849200	–0.26736200	0.96547600
H	–3.09639300	0.12136600	1.98279600
C	–4.36960300	1.67948800	0.29540200
C	–4.68468600	2.13265400	1.73042700
H	–3.85848000	1.95334800	2.40972600
H	–4.88444000	3.20050100	1.73507600
H	–5.56160000	1.61782900	2.10961300
O	–2.79087600	–1.65806900	1.07744600
C	–0.77344400	–0.58266100	0.87443400
C	–1.46421800	–1.83333600	1.24279300
O	–1.00272800	–2.85534900	1.64201200
C	0.49746400	–0.31453900	1.27154200
H	0.96252600	–1.00346400	1.96057400
H	0.85488300	0.70379600	1.26432200
O	–0.25209600	–1.67146900	–1.11617200
C	1.72263800	–0.88263900	–0.31209900

N	0.92016100	–1.41303100	–1.02042300
C	3.10863000	–0.51181300	–0.26793800
C	3.70653000	–0.13812000	0.92589900
C	3.85995300	–0.52504000	–1.43921100
C	5.03809700	0.21821100	0.95520700
H	3.13899500	–0.12855600	1.83997300
C	5.18981500	–0.17502400	–1.41319300
H	3.39770100	–0.81310300	–2.36774000
C	5.76802100	0.19625700	–0.21434600
H	5.50130900	0.50673100	1.88219300
H	5.77115300	–0.18763800	–2.31836500
Br	7.58380200	0.68091100	–0.17874400

TS3

C	1.36408100	2.57708800	0.33909000
C	1.67683500	3.89924500	0.18709100
O	0.14456000	4.69302900	–0.84632200
C	–0.84592400	2.70715200	–0.37510800
Br	–4.55585100	–2.04532500	0.17769300
C	3.54584900	–2.83311600	0.61668300
C	2.70642500	–1.68682800	1.02350000
C	2.06966300	–0.97795200	0.08530300
C	3.01730300	–2.41666300	–1.71577600
C	3.67282800	–3.11947300	–0.80716100
H	3.11576000	–2.65223800	–2.76649600
H	4.32251300	–3.93784300	–1.07107600
O	4.12925800	–3.51396200	1.43403200
C	2.66644800	–1.41691800	2.49466500
H	3.16799100	–0.48629500	2.74128000
H	1.65055200	–1.34027500	2.86072200
H	3.17251200	–2.22415800	3.00785100
C	2.55029700	–0.07603600	–2.22445600
H	3.61529000	0.07197800	–2.05372000
H	2.42829800	–0.32197400	–3.27662400
C	1.82768800	1.21940600	–1.88705100
H	2.27481200	2.03903300	–2.44207800
H	0.77557200	1.17563200	–2.16951600
C	1.95574400	1.42072100	–0.40113200
H	3.01894400	1.37784600	–0.14835500
C	1.27514700	0.25890300	0.30133000
H	0.29326900	0.11608500	–0.14547700
C	2.09620000	–1.30003900	–1.39347900
C	0.70170200	–1.74692400	–1.86322700
H	–0.01852900	–0.93559600	–1.84269100
H	0.76262300	–2.10276900	–2.88794400
H	0.32868100	–2.55403100	–1.24088900
O	1.05142100	0.70008700	1.62969900
C	0.95727400	2.04633400	1.64825900

O	0.60240400	2.63033100	2.62722900
N	–0.70365500	3.81885900	–0.77338400
C	–1.74125300	1.59816400	–0.24433800
C	–2.20976200	0.94648200	–1.38045600
C	–2.11487100	1.15374700	1.01819500
C	–3.05452600	–0.13325200	–1.25612500
H	–1.91839100	1.29349100	–2.35763100
C	–2.95235900	0.06755700	1.14435400
H	–1.74793700	1.65706900	1.89661400
C	–3.41322700	–0.56508700	0.00668100
H	–3.42839100	–0.63555200	–2.13085800
H	–3.24546700	–0.28125900	2.11871500
H	1.50828200	4.56872800	1.01350200
H	2.37347100	4.21554600	–0.56906700

TS4

C	0.58315600	3.53544300	0.13976100
C	0.71021500	2.17963500	0.71275400
C	1.82445500	1.47622800	0.48645400
C	2.75502300	3.30707300	–0.92257900
C	1.66772400	4.02729900	–0.70148600
H	3.54807700	3.69139100	–1.54918600
H	1.52994100	5.00694500	–1.12911600
O	–0.39619900	4.22192100	0.34352900
C	–0.46599300	1.70223500	1.50378000
H	–1.21654900	2.48168200	1.51621800
H	–0.89500800	0.80668900	1.06697000
H	–0.19058900	1.45996300	2.52363900
C	3.26326200	0.99529600	–1.51563400
H	2.45109100	1.10405400	–2.23256600
H	4.17373300	1.31801600	–2.01506100
C	3.36214000	–0.47018100	–1.11698500
H	3.44382900	–1.08063500	–2.01230900
H	4.25019900	–0.66103600	–0.51626200
C	2.12505500	–0.80855200	–0.33138000
H	1.25602200	–0.54311300	–0.93749200
C	2.07472400	0.07388500	0.90526800
H	3.04087500	0.01957100	1.40598700
C	2.99394800	1.96522600	–0.33852600
C	4.25831000	2.08906700	0.52613000
H	4.63592700	1.12319100	0.84333300
H	5.04283400	2.57162500	–0.04980600
H	4.06183400	2.69024500	1.40812400
O	1.14030900	–0.55608700	1.76886500
C	1.21057300	–1.88764300	1.57268800
O	0.73304800	–2.66085300	2.34587200
C	1.91738800	–2.14700200	0.30498500
O	1.56079200	–4.45552000	–1.08260900

C	0.02484000	–2.84476600	–0.67744500
N	0.49671600	–3.84992800	–1.10550800
C	–1.10745500	–1.98323600	–0.55574100
C	–1.93383200	–2.08256200	0.55891700
C	–1.36968900	–1.02861500	–1.53265900
C	–3.02760900	–1.25546000	0.68074700
H	–1.71334700	–2.80732500	1.32309400
C	–2.46199800	–0.19851600	–1.41227000
H	–0.72956900	–0.95247500	–2.39524100
C	–3.28160500	–0.32376900	–0.30748600
H	–3.67548900	–1.33281400	1.53589300
H	–2.67519100	0.53522600	–2.16925900
Br	–4.77209300	0.80512600	–0.14087700
C	2.66844500	–3.27792400	0.13641700
H	3.49044200	–3.28985400	–0.55776100
H	2.66711900	–4.02591900	0.91053500

TS5

C	1.35656600	–2.93155900	0.56083900
C	0.61437100	–1.85235600	0.95448600
O	0.78520600	–3.12209800	–1.55742800
C	–0.81688100	–1.84331300	–0.60836500
C	2.74418000	–2.81449800	0.13533100
C	3.30175400	–1.45842800	–0.04311000
C	2.61865500	–0.40898900	0.42742100
H	–0.20355300	–2.03587100	1.63771700
H	1.00752500	–3.93693100	0.72415200
O	3.41888400	–3.80618600	–0.03905700
C	4.66142600	–1.41942000	–0.66803400
H	5.39739300	–0.98377100	–0.00206400
H	4.96102500	–2.43271400	–0.90000900
H	4.66394200	–0.83058000	–1.57763100
C	0.31995400	0.62107300	0.69096700
H	0.02506600	0.43547400	–0.33837400
H	–0.58161300	0.55359300	1.29559600
C	0.90221200	2.02191500	0.73652900
H	0.17249500	2.72830900	0.35123700
H	1.15021700	2.33393900	1.74936700
C	2.13738000	1.98733700	–0.11644200
H	1.86898100	1.62097600	–1.11158400
C	3.12290600	0.99321100	0.46794300
H	3.27962200	1.25427300	1.51652100
C	1.27400900	–0.50356900	1.13159400
C	1.51414000	–0.38490600	2.64766400
H	1.92085600	0.58196200	2.92411000
H	0.57226100	–0.50698700	3.17557400
H	2.20015200	–1.15566300	2.98436400
O	4.34508200	1.27138300	–0.20340800

C	3.03443600	3.15547400	–0.29762100
C	4.38266700	2.57824500	–0.51741600
O	5.37175400	3.10919100	–0.90990100
C	2.80902200	4.45370900	–0.26602300
H	1.82573700	4.85738800	–0.08830200
H	3.62325100	5.14411200	–0.41719100
N	–0.24360600	–2.49590100	–1.43964400
C	–2.07597700	–1.15859100	–0.45326000
C	–2.80033000	–1.27608900	0.72302900
C	–2.56715700	–0.37047400	–1.48695500
C	–4.00329600	–0.61887400	0.86883400
H	–2.43440800	–1.89148400	1.52726900
C	–3.77134500	0.28282700	–1.34985600
H	–2.00431400	–0.27381300	–2.39977200
C	–4.47727700	0.15594500	–0.16962400
H	–4.56722600	–0.71429300	1.77993600
H	–4.15450600	0.88859700	–2.15219900
Br	–6.11638700	1.05612700	0.02484500

TS6

C	–0.14603000	–1.99429100	0.83724800
C	–1.31536400	–2.43577300	0.27620500
O	–0.83404300	–2.13092900	–1.81905600
C	1.07876700	–1.37722100	–0.86408400
C	–0.10988800	–0.69904200	1.50669700
C	–1.20599600	0.25610300	1.22733200
C	–2.34913600	–0.20641000	0.70833000
H	–1.43100100	–3.48540400	0.05722400
H	0.61082700	–2.70391900	1.13192300
O	0.79574900	–0.41313900	2.26246000
C	–0.94866900	1.66046700	1.67699400
H	0.00736700	1.69083100	2.18275600
H	–0.92586000	2.34615000	0.83719300
H	–1.71517800	2.01316300	2.35693900
C	–3.50714000	–1.94894200	–0.78572400
H	–2.93433100	–1.78619500	–1.69007900
H	–3.77380000	–3.00318000	–0.76189200
C	–4.74920600	–1.07917300	–0.86183400
H	–5.27402900	–1.28129100	–1.79125200
H	–5.44603800	–1.27473600	–0.04821900
C	–4.26865300	0.34209000	–0.79906500
H	–3.50631900	0.49043000	–1.56947500
C	–3.59759600	0.58606500	0.53885900
H	–4.29275200	0.27605900	1.32393100
C	–2.59403100	–1.67677100	0.42518600
C	–3.26777000	–2.27844000	1.67743100
H	–4.25366900	–1.85120500	1.83476800
H	–3.38819600	–3.35087800	1.55136700

H	–2.66713300	–2.09587800	2.56261400
O	–3.48323500	2.00120900	0.62551100
C	–5.18171300	1.51104600	–0.84074800
C	–4.50209700	2.56369200	–0.04831800
O	–4.74723900	3.72518300	0.02460700
C	–6.36290100	1.68757700	–1.39832000
H	–6.85128200	0.90632900	–1.95734700
H	–6.86826700	2.63460700	–1.29745400
N	0.29275500	–1.68016700	–1.70781500
C	2.36984500	–0.79402800	–0.65123900
C	3.13637500	–1.14767100	0.44915900
C	2.86099400	0.12930400	–1.56881900
C	4.38717200	–0.59799400	0.62651200
H	2.74835100	–1.83477800	1.17906900
C	4.11295400	0.67421100	–1.39915600
H	2.26193100	0.41033200	–2.41793600
C	4.86506800	0.30413100	–0.30106700
H	4.98267300	–0.86984700	1.48001400
H	4.49800900	1.38258800	–2.11136900
Br	6.57399000	1.05188100	–0.06387800

4

C	6.26921300	–0.43443900	–1.01859400
C	5.17678100	0.41633400	–0.49649600
C	4.13008500	–0.16120400	0.10367100
C	5.10962500	–2.42481100	–0.25809800
C	6.14474200	–1.87889000	–0.87392200
H	5.04011500	–3.49830900	–0.14980100
H	6.94537500	–2.46926900	–1.28838100
O	7.24017500	0.04682500	–1.56323100
C	5.36544000	1.88529400	–0.71140100
H	6.35879900	2.04940900	–1.10758200
H	4.64475400	2.28098000	–1.41966400
H	5.25211800	2.44352100	0.20933000
C	2.68537900	–2.18547100	–0.27764500
H	2.77801200	–2.13786600	–1.36127800
H	2.59143700	–3.23546900	–0.01131700
C	1.44293400	–1.41786800	0.14239100
H	0.58408300	–1.78929900	–0.41021200
H	1.22864800	–1.55015800	1.20025900
C	1.68691400	0.03590300	–0.14516900
H	1.87923700	0.17888100	–1.21088700
C	2.91227000	0.52961700	0.60258700
H	2.78361900	0.31976100	1.66286200
C	3.99693900	–1.64955000	0.34050600
C	4.01896900	–1.95489800	1.84701600
H	3.14270600	–1.57161200	2.35830500
H	4.03841100	–3.03076800	1.99589300

H	4.90296500	–1.52794700	2.30958900
O	2.85072500	1.94655100	0.45458600
C	0.69308200	1.08617100	0.27582300
C	1.58155900	2.32377200	0.30604000
O	1.24075200	3.45528100	0.21275400
C	–0.59709000	1.22166100	–0.48392400
H	–0.58871300	0.68030300	–1.42354900
H	–0.83411900	2.26525200	–0.68272500
O	0.29590600	0.84721900	1.63298000
C	–1.54576800	0.64850100	0.51653300
N	–1.02195000	0.47444900	1.65738200
C	–2.94860300	0.34150200	0.26860800
C	–3.51751800	0.62542200	–0.96262300
C	–3.73423800	–0.23415100	1.26073100
C	–4.84716300	0.34524900	–1.20739000
H	–2.92768200	1.07494400	–1.74428500
C	–5.05888800	–0.51883800	1.02829900
H	–3.29540700	–0.45724600	2.21823200
C	–5.60448800	–0.22557400	–0.20832300
H	–5.28460300	0.56947400	–2.16434000
H	–5.66456200	–0.96495600	1.79761100
Br	–7.41723000	–0.61600600	–0.53051900

5

C	–6.34319000	–0.61758300	0.07018100
C	–5.17542100	0.20088900	–0.32920900
C	–3.95899000	–0.35587900	–0.33188100
C	–4.90259900	–2.53285400	0.43654100
C	–6.11129500	–1.99821400	0.47241500
H	–4.74979000	–3.56351300	0.72517300
H	–6.97882900	–2.55443100	0.78744600
O	–7.46493500	–0.15722900	0.07498100
C	–5.50014800	1.61579400	–0.69274200
H	–6.57623800	1.72026900	–0.73243100
H	–5.11597300	2.31467900	0.04314500
H	–5.07867000	1.89191500	–1.65075300
C	–2.64941500	–1.92983900	1.13153000
H	–3.10781500	–1.56798800	2.04995200
H	–2.42955700	–2.98451700	1.27757000
C	–1.37037000	–1.14944500	0.88282800
H	–0.73286700	–1.20199600	1.76066100
H	–0.81326600	–1.57923400	0.05266600
C	–1.76220200	0.27138000	0.59038300
H	–2.32562600	0.66322700	1.43948900
C	–2.67714000	0.34753900	–0.61677100
H	–2.19364600	–0.12278700	–1.47379100
C	–3.68742900	–1.80792300	–0.00608900
C	–3.17817600	–2.54590100	–1.25494000

H	–2.20455600	–2.19224900	–1.57679400
H	–3.08367700	–3.60561000	–1.03521800
H	–3.87609600	–2.42672000	–2.07734000
O	–2.75426000	1.74039800	–0.89879500
C	–0.75882800	1.34374600	0.24281700
C	–1.64745600	2.35027900	–0.47174800
O	–1.42556400	3.49922100	–0.65397100
C	0.43793600	0.92919000	–0.59742700
H	0.63989000	1.62411300	–1.40923800
H	0.34060800	–0.06577900	–1.01792800
O	–0.19693000	1.93750500	1.40040700
C	1.51068900	1.02673400	0.43671100
N	1.12470900	1.59815800	1.49888300
C	2.88396300	0.57112700	0.26581000
C	3.29859400	0.04863600	–0.94874400
C	3.79599600	0.65967900	1.31127100
C	4.59938700	–0.37880200	–1.12662200
H	2.60937100	–0.02490100	–1.77388400
C	5.09344500	0.23572600	1.14649300
H	3.47769600	1.06456600	2.25675100
C	5.48399500	–0.28165000	–0.07520400
H	4.91666400	–0.78145300	–2.07245900
H	5.79711600	0.30445700	1.95756500
Br	7.25879300	–0.86497400	–0.30335300

6

C	4.42963600	–2.15926900	0.49324800
C	3.38630500	–1.22003400	0.96241600
C	2.42712300	–0.83495100	0.11390300
C	3.40920400	–2.22580400	–1.70680600
C	4.37936000	–2.60613200	–0.89265100
H	3.38253900	–2.57626700	–2.72927000
H	5.17041600	–3.26637700	–1.20817300
O	5.31203500	–2.54304000	1.23104100
C	3.52780800	–0.78102100	2.38591100
H	4.31780300	–1.35924200	2.84662000
H	3.78903000	0.27013400	2.45489600
H	2.61037000	–0.92253900	2.94303300
C	2.28189600	–0.14957200	–2.30310400
H	3.27571600	0.29450900	–2.31357400
H	2.09902600	–0.54652400	–3.29851900
C	1.26751600	0.93016000	–1.96609700
H	1.38177600	1.76976600	–2.64592200
H	0.24966700	0.56154400	–2.08123800
C	1.51512600	1.34886300	–0.54343800
H	2.54829900	1.69038100	–0.45106800
C	1.35982200	0.16406200	0.38789100
H	0.39016800	–0.30266000	0.22451100

C	2.29412700	–1.33343500	–1.30857900
C	1.00465300	–2.15603600	–1.46790100
H	0.11027300	–1.55041500	–1.36339200
H	0.98132000	–2.60147500	–2.45835500
H	0.96828400	–2.95307800	–0.73230400
O	1.31901600	0.74994200	1.68402400
C	0.85824200	1.99912800	1.60666300
O	0.66358900	2.67284900	2.56409900
C	0.65048700	2.38875300	0.15576400
O	0.03398900	4.19722300	–1.14640400
C	–0.78506000	2.37470600	–0.32092400
N	–1.04130100	3.36883900	–1.06547500
C	–1.77786500	1.31195700	–0.14532000
C	–1.96542400	0.68120000	1.07652700
C	–2.56386800	0.93584100	–1.22675300
C	–2.91186400	–0.31287600	1.21583300
H	–1.39849200	0.97504500	1.94400900
C	–3.51273600	–0.05462800	–1.09833700
H	–2.42100700	1.42487700	–2.17565400
C	–3.67464400	–0.67387600	0.12490700
H	–3.05665900	–0.79456300	2.16677200
H	–4.11502000	–0.34558400	–1.94112600
Br	–4.95986900	–2.03542900	0.30775800
C	0.94739700	3.84121000	–0.11827400
H	0.73666000	4.43606500	0.76825500
H	1.95186900	4.02926100	–0.47408500

7

C	–3.75531100	–2.91880200	0.08861700
C	–3.05987300	–1.80810700	0.77770100
C	–3.05588900	–0.59362700	0.21757500
C	–4.40045300	–1.42563900	–1.71025000
C	–4.39537100	–2.64138500	–1.19019900
H	–4.89207000	–1.23541500	–2.65426200
H	–4.86841800	–3.47808400	–1.67737400
O	–3.78650800	–4.03414500	0.56315700
C	–2.40236900	–2.18430100	2.06790700
H	–2.72798600	–3.17912100	2.34189500
H	–1.32067500	–2.19695500	1.97331100
H	–2.64973800	–1.49381000	2.86338000
C	–2.80204300	0.37015900	–2.09727300
H	–2.12433300	–0.41294800	–2.43253300
H	–3.37999400	0.68419900	–2.96278100
C	–1.98057900	1.52867700	–1.55741100
H	–1.25059700	1.84169800	–2.29869700
H	–2.61201100	2.38971000	–1.34797700
C	–1.30215100	1.04619800	–0.30602800
H	–0.70426800	0.16851000	–0.55716400

C	–2.32404700	0.60084600	0.72083500
H	–3.03567300	1.40774600	0.89710800
C	–3.77669700	–0.24456100	–1.06657800
C	–4.91988300	0.74322800	–0.78148700
H	–4.55814800	1.71236100	–0.45554600
H	–5.49606800	0.89982300	–1.68884400
H	–5.58205000	0.34842100	–0.01774400
O	–1.56555600	0.45468000	1.91567100
C	–0.53674200	1.30738700	1.90197300
O	0.15536600	1.49850100	2.84559600
C	–0.43727700	1.96345900	0.54156100
O	–0.10481400	4.03244200	–0.41922900
C	0.93871700	2.17816000	–0.02471600
N	1.04188600	3.31419000	–0.58365100
C	2.02980900	1.20915800	–0.08466600
C	1.90057400	–0.06635200	0.44307500
C	3.23193600	1.56445700	–0.68819400
C	2.93896600	–0.97311600	0.36906100
H	0.98991400	–0.38377200	0.92197700
C	4.27364500	0.67111300	–0.76647600
H	3.34331900	2.55440300	–1.09572400
C	4.11708200	–0.59578400	–0.23575200
H	2.82721500	–1.96022500	0.78170500
H	5.20038500	0.95491500	–1.23372600
Br	5.53945600	–1.82270700	–0.33985700
C	–0.85752200	3.42010000	0.61181600
H	–1.90799500	3.60653300	0.42828700
H	–0.56000800	3.84800500	1.56716000

8

C	–0.90386700	2.71444000	–0.04669100
C	–0.21513100	1.51233700	0.52044700
O	–0.58041200	2.66599800	–1.43486700
C	0.95448800	1.52205800	–0.43218800
C	–2.38930800	2.81222900	0.12139100
C	–3.14372200	1.62252200	0.52163900
C	–2.51748500	0.44072600	0.61870800
H	0.08151700	1.69892900	1.54689400
H	–0.46613700	3.62588000	0.35930700
O	–2.92253600	3.86887200	–0.10661300
C	–4.61497100	1.85065500	0.68160000
H	–4.79139100	2.91459400	0.77279000
H	–5.16842700	1.49745900	–0.18395000
H	–5.00864600	1.34268200	1.55021900
C	–0.79565800	–0.57229000	–0.85382500
H	–1.03669100	0.09532000	–1.67928600
H	0.25914200	–0.82158500	–0.93773100
C	–1.62119300	–1.84439900	–0.98226400

H	–1.49826200	–2.25114300	–1.98191100
H	–1.28212900	–2.60873000	–0.28639900
C	–3.06042900	–1.50783100	–0.71608600
H	–3.38194500	–0.74803600	–1.43470000
C	–3.21630600	–0.87330300	0.66246700
H	–2.76373100	–1.51635600	1.41091300
C	–1.02011400	0.18959800	0.47100600
C	–0.49141800	–0.65644000	1.63399800
H	–0.82692000	–1.68549500	1.61150000
H	0.59258200	–0.68179900	1.58351100
H	–0.77762300	–0.22334400	2.58876200
O	–4.61526000	–0.89833000	0.90191100
C	–4.13146500	–2.53166500	–0.64246600
C	–5.14783300	–1.96584200	0.27710700
O	–6.26218000	–2.32679800	0.47875500
C	–4.25455800	–3.71972800	–1.19965100
H	–3.49940100	–4.12324400	–1.85400300
H	–5.13005900	–4.31708000	–1.00194800
N	0.68496300	2.13701000	–1.51015000
C	2.25110700	0.87270900	–0.25439800
C	2.78392700	0.68665100	1.01183200
C	2.98355200	0.46265400	–1.36176600
C	4.02249900	0.10110900	1.17793100
H	2.24057400	1.01368900	1.88296800
C	4.21854700	–0.12538700	–1.20902300
H	2.57292700	0.60561900	–2.34692300
C	4.72676800	–0.30212600	0.06371400
H	4.43304600	–0.03590100	2.16283800
H	4.77971000	–0.44788000	–2.06857000
Br	6.41187600	–1.11150400	0.27949600

9

C	0.20746000	–0.79830400	0.92440700
C	–0.87209500	–1.71373200	0.41715200
O	–0.69730400	–1.67282600	–1.00160600
C	1.17277800	–0.92232800	–0.22669700
C	–0.20573400	0.65137200	1.04659700
C	–1.55030800	1.06763300	0.61081000
C	–2.51781800	0.14639500	0.50592400
H	–0.68090200	–2.73814200	0.73989600
H	0.63891100	–1.11679000	1.86627500
O	0.58007900	1.44244100	1.50523600
C	–1.73335300	2.54937600	0.48731500
H	–0.78395900	3.03608000	0.66550200
H	–2.09281000	2.81906300	–0.49792400
H	–2.45225900	2.92567300	1.20672000
C	–3.20765000	–2.27520400	–0.03654000
H	–2.82088800	–2.34268300	–1.04863100

H	–3.11711400	–3.26664600	0.40148200
C	–4.66260300	–1.85719000	–0.12884100
H	–5.19209500	–2.53326200	–0.79368400
H	–5.16409300	–1.89302500	0.83720000
C	–4.66058900	–0.45073700	–0.65490100
H	–4.07790400	–0.41687100	–1.57960600
C	–3.96618200	0.44812400	0.34941300
H	–4.42713700	0.26888700	1.32571100
C	–2.29171300	–1.33532100	0.77322900
C	–2.49289000	–1.59029600	2.26804800
H	–3.51912100	–1.39798200	2.56553700
H	–2.27090300	–2.62847600	2.50108400
H	–1.84892000	–0.95346900	2.86829600
O	–4.32013900	1.76705300	–0.03894300
C	–5.90461600	0.32908900	–0.87367900
C	–5.53301300	1.74146000	–0.62198400
O	–6.13829600	2.73435800	–0.86888500
C	–7.12834300	–0.04010400	–1.19548800
H	–7.38770500	–1.07331900	–1.35838300
H	–7.90344900	0.70211700	–1.29905200
N	0.60932100	–1.38323100	–1.26626900
C	2.58198500	–0.54726200	–0.22681400
C	3.20399300	–0.10878200	0.93132600
C	3.32877700	–0.65381800	–1.39549600
C	4.54542600	0.21843100	0.93026500
H	2.63825800	0.00433400	1.83909500
C	4.66467800	–0.32937100	–1.40856500
H	2.85086300	–0.99649600	–2.29726200
C	5.26286900	0.10398600	–0.23976700
H	5.02231500	0.56065700	1.83191500
H	5.23792000	–0.41231700	–2.31535300
Br	7.09270500	0.54761700	–0.25073200
